# Clinical vs. pathological staging in oral squamous cell carcinoma: frequency of misjudgment, associated risk factors, and impact on survival outcomes

**DOI:** 10.1007/s00784-026-07275-y

**Published:** 2026-11-14

**Authors:** Ann-Kristin Struckmeier, Martin Gosau, Christian Betz, Waldemar Wilczak, Ralf Smeets

**Affiliations:** 1 Department of Oral and Maxillofacial Surgery, University Medical Center Hamburg- Eppendorf, Martinistraße 52, 20246 Hamburg, Germany; 2 Department of Otorhinolaryngology, University Medical Center Hamburg- Eppendorf, Hamburg, Germany; 3 Department of Pathology, University Medical Center Hamburg-Eppendorf, Hamburg, Germany; 4 Department of Oral and Maxillofacial Surgery, Division of Regenerative Orofacial Medicine, University Medical Center Hamburg-Eppendorf, Hamburg, Germany

**Keywords:** Oral squamous cell carcinoma, Head and neck squamous cell carcinoma, Staging, Neck dissection, Survival

## Abstract

**Objectives:**

Accurate staging of oral squamous cell carcinoma (OSCC) is crucial for prognosis and treatment planning. This study evaluates clinical staging accuracy, identifies factors contributing to discrepancies, and examines their impact on survival, particularly with the rising role of neoadjuvant immunotherapy.

**Materials and methods:**

A retrospective analysis of 545 OSCC patients assessed concordance between clinical and pathological staging. Logistic regression identified risk factors for misjudgment, while Kaplan-Meier curves and Cox proportional hazards models evaluated survival outcomes.

**Results:**

T stage concordance was observed in 315 patients (57.79%), with 125 (22.93%) upstaged and 104 (19.08%) downstaged. N stage concordance occurred in 319 patients (58.53%), with 159 (29.17%) upstaged and 67 (12.29%) downstaged. The highest T stage concordance was seen in cT4a (76.55%) and cT1 (68.32%), while cN0 showed the highest N stage accuracy (74.21%). Histopathological factors, including grading, perineural invasion, and lymphatic invasion, were significantly associated with staging misjudgment. Both N-stage upstaging and downstaging were independently associated with worse overall survival, whereas T-stage discordance was not significantly associated with overall survival.

**Conclusions:**

Significant discrepancies exist between clinical and pathological staging in OSCC. Histopathological features such as grading, perineural invasion, and lymphatic invasion were associated with staging discordance and may help identify patients at increased risk of clinical understaging.

**Clinical relevance:**

These findings highlight the need for enhanced staging strategies to optimize patient outcomes.

**Supplementary Information:**

The online version contains supplementary material available at https://doi.org/10.1007/s00784-026-07275-y.

## Introduction

Accurate clinical staging of head and neck squamous cell carcinoma (HNSCC), particularly oral squamous cell carcinoma (OSCC), is essential for guiding treatment decisions and predicting patient outcomes. Clinical staging is based on a comprehensive evaluation that combines physical examination and radiologic imaging to determine the extent of the primary tumor, assess regional lymph node involvement, and detect potential distant metastases. Tumors are then classified according to the tumor, node, metastasis (TNM) system. However, discrepancies between clinical staging (cTNM) and staging determined after histopathological assessment (pTNM) are frequently reported [1–5], which can lead to variations in treatment planning and prognostication. While physical examination may be limited by factors such as obesity, tumor localization, pain, or trismus [4], staging accuracy may also be influenced by examiner experience, recent biopsy-related changes, and the imaging modality used. Imaging modalities such as computed tomography (CT) and magnetic resonance imaging (MRI) may themselves face challenges, including the assessment of bone invasion and lymph node metastases [6, 7].

Discrepancies between clinical and pathological staging are particularly relevant when treatment decisions must be made without definitive histopathological assessment. With the increasing integration of neoadjuvant therapy and de-escalated neck management strategies, such as sentinel lymph node biopsy (SLNB), into the management of OSCC [8–10], precise clinical staging has become even more critical. Misjudgment, particularly in cases of overdiagnosis, may lead to overtreatment, thereby exposing patients to unnecessary toxicities and costs. Furthermore, underestimating tumor size or lymph node metastases can result in inadequate treatment, potentially compromising patient outcomes or necessitating multiple surgeries and adjuvant therapies, which may lead to treatment delays and adversely affect quality of life [11, 12]. As a result, accurate staging is not only important for selecting the most appropriate treatment but also for predicting patient prognosis and guiding clinical decisions, including eligibility for clinical trials and postoperative surveillance.

This study aims to evaluate the accuracy of clinical staging in OSCC by examining discrepancies between clinical and pathological staging, identifying risk factors for misjudgment, and assessing the impact of these discrepancies on patient survival outcomes.

## Materials and methods

### Study design and participants

Patients diagnosed with primary OSCC who met the criteria for surgical management, including radical tumor resection and neck dissection, as per oncology guidelines, were included in the study. Only patients with complete data on both clinical and pathological T, N, and M staging were considered. Consequently, patients with Tx, Nx, or Mx classifications were excluded, resulting in the exclusion of 281 patients from an initial cohort of 826. Treatment followed the prevailing German guidelines and was conducted at a high-volume center between January 1, 2010, and December 31, 2020. The last follow-up date was November 30, 2024.

Exclusion criteria included recurrent or secondary OSCC, non-OSCC malignancies, prior treatment (surgery or radiotherapy) in the head and neck region, neoadjuvant therapy, and squamous cell carcinoma of the lip. Patients who underwent sentinel lymph node biopsy were also excluded.

A comprehensive set of parameters was recorded and analyzed, including age, sex, tumor localization, clinical and pathological TNM classification, depth of invasion, histological grading, and the presence of perineural, lymphatic, and vascular invasion. All data were extracted from hospital medical records. During the study period, the TNM classification system underwent revision. To ensure consistency in our findings [13], patients initially classified under the 7th edition of the TNM classification (before 2017) were reclassified according to the 8th edition. Consequently, all patients in this study were categorized based on the 8th TNM classification.

The study design and methodology were approved by the Ethics Committee of the Ärztekammer Hamburg (Ethics vote: 2024-101379-BO-ff). In compliance with national and institutional regulations, written informed consent was not required.

This manuscript was prepared in accordance with the STROBE guidelines.

### Staging process

Clinical staging was based on the integrated assessment of physical examination and contrast-enhanced CT. Physical examination was routinely performed by an experienced consultant in oral and maxillofacial surgery. Contrast-enhanced CT represented the standard imaging modality during the study period in accordance with the German S3 guideline for OSCC [14]. CT scans were performed from the head to at least the upper abdomen to ensure comprehensive assessment.

Surgical specimens were obtained intraoperatively and subsequently oriented by the surgeon before formalin fixation. Specimen processing was conducted in the Department of Pathology, where cross-sectioning was performed, and tissues were embedded in paraffin. Paraffin blocks were sectioned into 4-µm-thick slices, which were then analyzed by senior pathologists.

### Therapy regimen

All treatment decisions were made by the interdisciplinary tumor board and followed the recommendations of the German S3 guideline for OSCC [14]. Surgical treatment was performed with oncologic intent, aiming for complete resection of the primary tumor with microscopically negative margins. Neck dissection was performed according to clinical nodal status, tumor localization, and the individual risk profile. In clinically node-negative patients, an ipsilateral selective neck dissection of levels I–III or I-IV was performed. For midline tumors or tumors approaching the midline, bilateral selective neck dissection was performed. In patients with clinically suspected or confirmed ipsilateral lymph node metastases, ipsilateral modified radical neck dissection was performed and complemented by contralateral selective neck dissection. In cases with contralateral lymph node metastases, bilateral modified radical neck dissection was performed. Reconstructive procedures were performed when required to restore function and form.

Patients with distant metastases were included in case of singular and technically resectable metastases, for example by video-assisted thoracoscopic surgery in the case of pulmonary metastasis. Due to the limited metastatic disease, relatively young age, and good general condition of these patients, the interdisciplinary tumor board recommended radical resection of the primary tumor and neck dissection with curative or locally curative intent as part of an individualized treatment concept.

The indication for adjuvant radiotherapy or radiochemotherapy was determined according to the German S3 guideline for the diagnosis and treatment of OSCC [14] and was discussed individually in the interdisciplinary tumor board. According to these recommendations, postoperative radiotherapy or radiochemotherapy was considered in patients with adverse pathological risk factors, including advanced T category (T3/T4), close or positive resection margins, perineural invasion, vascular invasion, and/or lymph node involvement. In patients with an indication for postoperative radiotherapy and high-risk features such as a resection margin < 5 mm and/or extranodal extension, cisplatin-based radiochemotherapy was recommended.

### Statistical analysis

Baseline data were analyzed descriptively. The concordance between clinical and pathological T and N stages was evaluated by calculating the percentages of patients with accurate staging (cT = pT, cN = pN), underdiagnosis (cT < pT, cN < pN), and overdiagnosis (cT > pT, cN > pN).

Correlation analysis was performed using the chi-square test. To evaluate risk factors for staging misjudgment, logistic regression analysis was conducted, followed by multivariable analysis incorporating factors with *p* < 0.05 in the univariable analysis. Recurrence-free survival (RFS), local recurrence-free survival (LRFS), progression-free survival (PFS), and overall survival (OS) were estimated using the Kaplan-Meier method. RFS assessed postoperative recurrence, whereas PFS was additionally calculated as a broader disease-control endpoint to account for patients with known metastatic or residual disease. Survival outcomes were compared using the log-rank test. Prognostic factors for survival were identified through univariate Cox regression analysis, followed by multivariable Cox regression analysis including factors with *p* < 0.05 in the univariable analysis.

Statistical analyses were performed using the Statistical Package for the Social Sciences (SPSS), version 28.0 (Chicago, IL, USA). The date of analysis was December 20, 2024.

A p-value of < 0.05 was considered statistically significant.

## Results

### Clinicopathological characteristics

The study cohort comprised 545 patients diagnosed with OSCC, with a gender distribution of 60.00% males and 40.00% females. The age distribution was nearly balanced, with 51.93% of patients under 65 years of age and 48.07% aged 65 years or older.

The majority of tumors were staged as either pT1 (31.93%) or pT4a (30.28%), with intermediate stages pT2 (25.32%) and pT3 (12.11%) accounting for the remainder. Advanced T4b tumors were rare, accounting for only 0.37%. Regarding nodal involvement, 60.18% of patients were classified as pN0, 14.50% as pN1, 2.95% as pN2a, 8.62% as pN2b, 4.77% as pN2c, 0.37% as pN3a, and 8.62% as pN3b. Distant metastases were infrequent, with 99.08% classified as M0 and 0.92% as M1.

Regarding grading, moderately differentiated tumors (G2) comprised 66.24%, poorly differentiated tumors (G3) 22.02%, and well-differentiated tumors (G1) 6.42%. Lymphatic invasion was observed in 17.61% of cases, vascular invasion in 4.22%, and perineural invasion in 22.57% of the patients. The majority of resections achieved clear margins (R0) in 96.70% of patients.

Recurrence was observed in 20.18% of patients, and 30.83% had died by the time of data analysis. Local recurrence was observed in 95 patients (17.43%), and regional/neck recurrence was observed in 18 patients (3.30%). Adjuvant radiation therapy was administered to 53.03% of patients, while 21.10% received chemotherapy. Distant metastases during the follow-up period occurred in 8.99% of patients. Further details are provided in Table 1.

**Table 1:** Clinicopathological characteristics

Variable	Category	*n* (%)
Sex	**Male**	327 (60.00)
	**Female**	218 (40.00)
Age at surgery	**< 65 years**	283 (51.93)
	**≥ 65 years**	262 (48.07)
Pathological T stage	**T1**	174 (31.93)
	**T2**	138 (25.32)
	**T3**	66 (12.11)
	**T4a**	165 (30.28)
	**T4b**	2 (0.37)
Pathological N stage	**N0**	328 (60.18)
	**N1**	79 (14.50)
	**N2a**	16 (2.94)
	**N2b**	47 (8.62)
	**N2c**	26 (4.77)
	**N3a**	2 (0.37)
	**N3b**	47 (8.62)
Depth of invasion	**≤ 5 mm**	205 (37.61)
	**6–10 mm**	107 (19.63)
	**> 10 mm**	183 (33.58)
	**DOIx**	50 (9.17)
Extranodal extension	**ENE(+)**	126 (23.12)
	**ENE(-)**	404 (74.13)
	**ENEx**	15 (2.75)
M stage (at first presentation)	**M0**	540 (99.08)
	**M1**	5 (0.92)
Grading	**G1**	35 (6.42)
	**G2**	361 (66.24)
	**G3**	120 (22.02)
	**Gx**	29 (5.32)
Lymphatic invasion	**L0**	449 (82.39)
	**L1**	96 (17.61)
Vascular invasion	**V0**	522 (95.78)
	**V1**	23 (4.22)
Perineural invasion	**Pn0**	422 (77.43)
	**Pn1**	123 (22.57)
Resection margin	**R0**	527 (96.70)
	**R1**	15 (2.75)
	**R2**	3 (0.55)
Recurrence	**No**	435 (79.82)
	**Yes**	110 (20.18)
Death	**No**	377 (69.17)
	**Yes**	168 (30.83)
Distant metastases during follow-up	**No**	496 (91.01)
	**Yes**	49 (8.99)
Localization	**Tongue**	139 (25.50)
	**Floor of the mouth**	152 (27.89)
	**Lower jaw**	107 (19.63)
	**Upper jaw**	44 (8.07)
	**Hard palate**	61 (11.19)
	**Buccal plane**	41 (7.71)
Adjuvant Therapy: Radiation	**No**	256 (46.97)
	**Yes**	289 (53.03)
Adjuvant Therapy: Chemotherapy	**No**	430 (78.90)
	**Yes**	115 (21.10)

*M stage* distant metastases staging, *N stage* nodal staging, *T stage* tumor staging

The median follow-up period, calculated from diagnosis to last contact or death, was 40 months, with a mean follow-up of 46.05 months and a range of 0 to 157 months.

### Concordance between clinical and pathological T staging

Next, an analysis of concordance between clinical and pathological T staging in OSCC was conducted. Among patients clinically staged as T1, 68.32% (*n* = 110) were diagnosed as pT1. However, 31.68% (*n* = 51) of cases were underdiagnosed, with most being reclassified as pT2 (22.98%, *n* = 37), and smaller proportions being upstaged to pT3 (2.48%, *n* = 4) or pT4a (6.21%, *n* = 10).

For cT2 patients, concordance between clinical and pathological staging was achieved in 39.90% (*n* = 77) of cases. Overdiagnosis was observed in 29.02% (*n* = 56), with all of them being downgraded to pT1. Underdiagnosis occurred in 31.08% (*n* = 60), leading to reclassification as pT3 in 16.06% (*n* = 31) and as pT4a in 14.51% (*n* = 28). One patient (0.52%) was later staged as pT4b.

In patients clinically staged as T3, the concordance rate was 39.53% (*n* = 17). Overdiagnosis occurred in 30.23% (*n* = 13), with cases being downgraded to pT2 (20.93%, *n* = 9) and pT1 (9.30%, *n* = 4). Underdiagnosis accounted for 30.23% (*n* = 13), with all of them being upstaged to stage pT4a.

For cT4a staging, the concordance was highest at 76.55% (*n* = 111). A small proportion (0.69%, *n* = 1) was underdiagnosed, while 22.76% (*n* = 33) were overdiagnosed and subsequently downgraded to pT3 (9.66%, *n* = 14), pT2 (10.34%, *n* = 15), or pT1 (2.76%, *n* = 4). All cases clinically staged as cT4b (*n* = 2) were diagnosed as pT4a after histopathological assessment and thereby misdiagnosed.

Overall, T staging concordance was achieved in 315 patients (57.79%), while 125 patients (22.93%) were upstaged, and 104 patients (19.08%) were downstaged. Please see Table 2 for further details.

**Table 2:** Concordance between clinical and pathological T staging

	pT1	pT2	pT3	pT4a	pT4b	Correct staging, *n* (%)	Underdiagnosed, *n* (%)	Overdiagnosed, *n* (%)	Misdiagnosed, *n* (%)
cT1	110 (68.32)	37 (22.98)	4 (2.48)	10 (6.21)	0 (0.00)	110 (68.32)	51 (31.68)	0 (0.00)	51 (31.68)
cT2	56 29.02)	77 (39.90)	31 (16.06)	28 (14.51)	1 (0.52)	77 (39.90)	60 (31.08)	56 (29.02)	116 (60.10)
cT3	4 (9.30)	9 (20.93)	17 (39.53)	13 (30.23)	0 (0.00)	17 (39.53)	13 (30.23)	13 (30.23)	26 (60.47)
cT4a	4 (2.76)	15 (10.34)	14 (9.66)	111 (76.55)	1 (0.69)	111 (76.55)	1 (0.69)	33 (22.76)	34 (23.45)
cT4b	0 (0.00)	0 (0.00)	0 (0.00)	2 (100.00)	0 (0.00)	0 (0.00)	0 (0.00)	2 (100.00)	2 (100.00)

*cT* clinical tumor staging, *pT* pathological tumor staging

### Concordance between clinical and pathological N staging

For patients clinically staged as N0, 74.21% (*n* = 282) were identified as pN0 after histopathological assessment. However, 25.79% (*n* = 98) were underdiagnosed, with nodal involvement upstaged to pN1 (11.58%, *n* = 44), pN2a (1.84%, *n* = 7), pN2b (6.32%, *n* = 24), pN2c (2.11%, *n* = 8), or pN3b (3.95%, *n* = 15).

Among patients clinically staged as N1, only 30.77% (*n* = 20) were confirmed as pN1. Overdiagnosis occurred in 23.08% (*n* = 15), with cases reclassified as pN0. Underdiagnosis was more common, affecting 46.15% (*n* = 30), with upstaging to higher nodal categories, such as pN2a (4.62%, *n* = 3), pN2b (18.46%, *n* = 12), pN2c (7.69%, *n* = 5), pN3a (1.54%, *n* = 1), or pN3b (13.85%, *n* = 9).

In patients clinically staged as cN2a, only 25% (*n* = 3) were classified as pN2a. Overdiagnosis was observed in 33.33% (*n* = 4), with cases downgraded to pN0 or pN1. Underdiagnosis affected 41.67% (*n* = 5), with upstaging to pN2b or pN3b.

For cN2b patients, the concordance rate was particularly low at 11.54% (*n* = 6). Underdiagnosis occurred in 40.38% (*n* = 21), whereas overdiagnosis was observed in 48.08% (*n* = 25). Overall, this group demonstrated an 88.46% staging discordance rate.

Among cN2c patients, only 19.35% (*n* = 6) were confirmed as pN2c. Overdiagnosis was common, occurring in 64.52% (*n* = 20), with patients downgraded to pN0 or pN1. Underdiagnosis affected 16.13% (*n* = 5), with upstaging to pN3b.

Among patients clinically staged as N3a, 33.33% (*n* = 1) were correctly classified as pN3a, whereas 66.67% (*n* = 2) were overdiagnosed and subsequently classified as pN0. In the cN3b group, one patient was correctly staged, while the other was downstaged to pN1 following histopathological assessment.

In summary, overall concordance between clinical and pathological N staging was observed in 319 patients (58.53%). However, 159 patients (29.17%) were upstaged, and 67 patients (12.29%) were downstaged. Please refer to Table 3.

**Table 3:** Concordance between clinical and pathological N staging

	pN0	pN1	pN2a	pN2b	pN2c	pN3a	pN3b	Correct staging, *n* (%)	Under-diagnosed, *n* (%)	Over-diagnosed, *n* (%)	Mis-diagnosed, *n* (%)
cN0	282 (74.21)	44 (11.58)	7 (1.84)	24 (6.32)	8 (2.11)	0 (0.00)	15 (3.95)	282 (74.21)	98 (25.79)	0 (0.00)	98 (25.79)
cN1	15 (23.08)	20 (30.77)	3 (4.62)	12 (18.46)	5 (7.69)	1 (1.54)	9 (13.85)	20 (30.77)	30 (46.15)	15 (23.08)	45 (69.23)
cN2a	3 (25.00)	1 (8.33)	3 (25.00)	2 (16.67)	0 (0.00)	0 (0.00)	3 (25.00)	3 (25.00)	5 (41.67)	4 (33.33)	9 (75.00)
cN2b	14 (26.92)	9 (17.31)	2 (3.85)	6 (11.54)	7 (13.46)	0 (0.00)	14 (26.92)	6 (11.54)	21 (40.38)	25 (48.08)	46 (88.46)
cN2c	12 (38.71)	4 (12.90)	1 (3.23)	3 (9.68)	6 (19.35)	1 (3.23)	4 (12.90)	6 (19.35)	5 (16.13)	20 (64.52)	25 (80.65)
cN3a	2 (66.67)	0 (0.00)	0 (0.00)	0 (0.00)	0 (0.00)	1 (33.33)	0 (0.00)	1 (33.33)	0 (0.00)	2 (66.67)	2 (66.67)
cN3b	0 (0.00)	1 (50.00)	0 (0.00)	0 (0.00)	0 (0.00)	0 (0.00)	1 (50.00)	1 (50.00)	0 (0.00)	1 (50.00)	1 (50.00)

*cN* clinical nodal staging, *pN* pathological nodal staging

### Association of misdiagnosis with clinicopathological characteristics

The association between clinicopathological characteristics and the concordance of clinical and pathological staging in OSCC was analyzed to explore factors influencing diagnostic accuracy. The chi-square (χ²) test was used to assess the statistical significance of differences among various variables.

In terms of sex, no significant association was observed with staging accuracy (*p* = 0.864). The concordance rates between clinical and pathological staging were similar for males (58.72%) and females (57.34%), with comparable rates of upstaging and downstaging. For age, no significant association with staging concordance was found (*p* = 0.964). The concordance rate was 57.80% in patients under 65 years and 58.78% in those aged 65 years and older. The rates of upstaging and downstaging were also similar across these age groups. Tumor grading showed a significant relationship with staging concordance (*p* = 0.029). Well-differentiated tumors (G1) had the highest concordance rate at 65.71%, while poorly differentiated tumors (G3) had the lowest rate at 51.67%. Upstaging was most common in G3 tumors (32.50%), while downstaging occurred more frequently in G1 tumors (25.71%). The presence of lymphatic invasion also had a significant association with staging accuracy (*p* = 0.034). The concordance rates were similar for patients without lymphatic invasion (L0) and those with lymphatic invasion (L1) at 57.38% and 56.25%, respectively. However, upstaging was notably higher in L1 cases (32.39%) compared to L0 cases (22.14%).

Vascular invasion did not significantly impact staging accuracy (*p* = 0.210). The concordance rate for patients without vascular invasion (V0) was 57.06%, while those with vascular invasion (V1) showed a similar rate of 56.52%. Although upstaging occurred more frequently in V1 cases (39.13%) compared to V0 cases (23.59%), the difference was not statistically significant. Perineural invasion demonstrated a significant association with staging concordance (*p* = 0.003). Patients without perineural invasion (Pn0) had a higher concordance rate of 59.51%, compared to 49.59% in those with perineural invasion (Pn1). Additionally, upstaging was more frequent in Pn1 cases (35.77%) than in Pn0 cases (20.65%).

Regarding resection margin status, there was no significant association with staging accuracy (*p* = 0.611). Patients with clear surgical margins had a concordance rate of 57.69%, while those with microscopically positive resection margins showed a slightly higher rate of 66.67%. Patients with macroscopically positive margins had 100% concordance, although this category involved only a small sample size.

Similarly, distant metastases were not significantly associated with concordance (*p* = 0.273). Patients without metastases had a concordance rate of 57.46%, while those with metastases exhibited a higher rate of 65.31%.

Tumor localization did not significantly affect staging accuracy (*p* = 0.347). The highest concordance rate was observed in tumors of the lower jaw (67.29%), while tongue tumors had the lowest concordance rate at 54.68%. Interestingly, tongue tumors also showed the highest rate of upstaging (28.78%).

In terms of adjuvant therapy, staging concordance did not have a significant impact on chemotherapy (*p* = 0.334). Patients who received chemotherapy had a concordance rate of 56.52%, while those who did not receive chemotherapy had a slightly higher rate of 58.60%. However, radiation therapy showed a significant association with staging accuracy (*p* = 0.018). Patients who received radiation had higher rates of upstaging (26.99%) and lower rates of downstaging (15.22%) compared to those who did not receive radiation, who had rates of 18.75% for upstaging and 22.66% for downstaging.

Recurrence also did not show a significant relationship with staging concordance (*p* = 0.431). The concordance rate was 57.70% in patients without recurrence, slightly lower than the 60.00% observed in those with recurrence.

Survival status did not show a significant association with staging accuracy (*p* = 0.198). Patients who survived had a concordance rate of 59.68%, while those who had died exhibited a slightly lower rate of 54.76%. Please refer to Table 4.

**Table 4:** Association of concordance between clinical and pathological T staging with clinicopathological characteristics

Variable	Category	Concordance of cT and pT classification			
		Correct staging, n (%)	Upstaging, n (%)	Downstaging, n (%)	p value
Sex	**Male**	192 (58.72)	73 (22.32)	62 (18.92)	0.864
	**Female**	125 (57.34)	53 (24.31)	40 (18.35)	
Age at surgery	**< 65 years**	163 (57.80)	65 (23.05)	54 (19.15)	0.964
	**≥ 65 years**	152 (57.57)	60 (22.90)	52 (18.32)	
Grading	**G1**	23 (65.71)	3 (8.57)	9 (25.71)	0.029*
	**G2**	213 (59.00)	78 (21.61)	70 (19.39)	
	**G3**	62 (51.67)	39 (32.50)	19 (15.83)	
Lymphatic invasion	**L0**	241 (57.38)	93 (22.14)	86 (20.48)	0.034*
	**L1**	54 (56.25)	31 (32.39)	11 (11.46)	
Vascular invasion	**V0**	283 (57.06)	117 (23.59)	96 (19.35)	0.210
	**V1**	13 (56.52)	9 (39.13)	1 (4.35)	
Perineural invasion	**Pn0**	219 (59.51)	76 (20.65)	73 (19.84)	0.003*
	**Pn1**	61 (49.59)	44 (35.77)	18 (14.63)	
Resection margin	**R0**	304 (57.69)	123 (23.34)	100 (18.98)	0.611
	**R1**	10 (66.67)	3 (20.00)	0 (0.00)	
	**R2**	3 (100.00)	0 (0.00)	0 (0.00)	
Recurrence	**No**	251 (57.70)	98 (22.53)	86 (19.77)	0.431
	**Yes**	66 (60.00)	28 (25.45)	16 (14.55)	
Death	**No**	225 (59.68)	89 (23.61)	63 (16.71)	0.198
	**Yes**	92 (54.76)	37 (22.02)	39 (23.21)	
Distant metastases during follow-up	**No**	285 (57.46)	114 (22.98)	97 (19.56)	0.273
	**Yes**	32 (65.31)	12 (24.49)	5 (10.20)	
Localization	**Tongue**	76 (54.68)	40 (28.78)	23 (16.55)	0.347
	**Floor of the mouth**	84 (55.26)	35 (23.03)	33 (21.71)	
	**Lower jaw**	72 (67.29)	22 (20.56)	13 (12.15)	
	**Upper jaw**	25 (56.82)	10 (22.73)	9 (20.45)	
	**Hard palate**	33 (54.10)	13 (21.31)	15 (24.59)	
	**Buccal plane**	27 (64.29)	6 (14.29)	9 (21.43)	
Adjuvant therapy: Chemotherapy	**No**	252 (58.60)	94 (21.86)	84 (19.53)	0.334
	**Yes**	65 (56.52)	32 (27.83)	18 (15.65)	
Adjuvant therapy: Radiation	**No**	150 (58.59)	48 (18.75)	58 (22.66)	0.018*
	**Yes**	167 (57.79)	78 (26.99)	44 (15.22)	

A p value < 0.05 was considered statistically significant. Statistically significant differences are marked with an asterisk

### Univariate and multivariate analysis of risk factors for upstaging T stage

The univariate and multivariate analyses in Table S1 identified several factors associated with T stage upstaging following histopathological evaluation in OSCC.

No significant association was found between age or sex and T stage upstaging. Regarding clinical T stage, T2 was significantly associated with upstaging in the univariate analysis (OR = 1.824, *p* = 0.013), but not in the multivariate analysis (OR = 1.273, *p* = 0.377). T4a was significantly less likely to be upstaged in both analyses (univariate: OR = 0.020, *p* < 0.001; multivariate: OR = 0.012, *p* < 0.001). No significant associations were found for T3 or T4b.

Among grading, G3 tumors were significantly associated with upstaging in both analyses (univariate: OR = 4.823, *p* = 0.015; multivariate: OR = 4.483, *p* = 0.030). Perineural invasion was also significantly associated with upstaging (univariate: OR = 2.079, *p* = 0.002; multivariate: OR = 2.459, *p* = 0.002).

Other factors such as pathological N stage, lymphatic invasion, vascular invasion, and resection margin status did not show significant associations with T stage upstaging.

### Univariate and multivariate analysis of risk factors for downstaging T stage

The univariate analysis in Table S2 examined clinicopathological factors associated with downstaging of T stage following histopathological assessment in OSCC.

Age and sex did not show significant associations with T stage downstaging, with p-values of 0.789 and 0.696, respectively. However, clinical T stage demonstrated strong associations with downstaging. Patients with T2, T3, and T4a tumors had significantly higher odds of downstaging compared to T1, with ORs of 78.158 (*p* < 0.001), 90.588 (*p* < 0.001), and 32.703 (*p* < 0.001), respectively.

Regarding pathological N stage, no significant associations were found between N stage (pN0 vs. other stages) and downstaging, with p-values ranging from 0.266 to 0.423. Similarly, grading, lymphatic invasion, vascular invasion, perineural invasion, and resection margin did not show significant associations with downstaging, with p-values ranging from 0.113 to 0.675.

### Univariate and multivariate analysis of risk factors for upstaging N stage

The univariate and multivariate analyses in Table S3 explored the risk factors for upstaging N stage following histopathological assessment in OSCC.

Age and sex were not significantly associated with N stage upstaging, with p-values of 0.848 and 0.811, respectively. However, clinical N stage showed strong associations. Patients clinically staged as N1 had a significantly higher likelihood of upstaging compared to N0 in both the univariate (OR = 4.316, *p* < 0.001) and multivariate analyses (OR = 2.361, *p* = 0.029). Similarly, N2b patients had an increased likelihood of upstaging, with univariate and multivariate ORs of 9.592 (*p* < 0.001) and 5.667 (*p* = 0.002), respectively. Other clinical N stages, including N2a, N2c, and N3a/b, did not show significant associations with upstaging.

Pathological T stage was also a significant predictor. T2 and T3 tumors were associated with higher chances of N stage upstaging, with univariate ORs of 2.677 (*p* < 0.001) and 5.786 (*p* < 0.001), respectively. However, T4a and T4b were not significantly associated with N stage upstaging in the multivariate analysis, with p-values of 0.156 and 0.99, respectively.

In terms of grading, G2 and G3 tumors were associated with increased risk of upstaging in the univariate analysis, with ORs of 11.239 (*p* = 0.018) and 24.537 (*p* = 0.002), respectively. However, in the multivariate analysis, neither G2 nor G3 showed a significant association with N stage upstaging (both *p* = 0.998).

Lymphatic invasion was a strong risk factor for N stage upstaging, with both univariate (OR = 7.702, *p* < 0.001) and multivariate (OR = 6.017, *p* < 0.001) analyses showing a significant association. Similarly, perineural invasion was associated with upstaging in both analyses (univariate: OR = 7.541, *p* < 0.001; multivariate: OR = 1.838, *p* = 0.029). While vascular invasion was associated with increased risk of upstaging in the univariate analysis (OR = 5.860, *p* < 0.001), it did not show a significant association in the multivariate analysis (*p* = 0.519). Resection margin was not a significant predictor of N stage upstaging (*p* = 0.415).

### Univariate and multivariate analysis of risk factors for downstaging N stage

The univariate and multivariate analyses in Table S4 explored risk factors associated with downstaging of N stage following histopathological assessment in OSCC.

Age and sex were not significantly associated with N stage downstaging, with p-values of 0.301 and 0.519, respectively. In terms of clinical N stage, no significant associations were found between any clinical N stage (N1, N2a, N2b, N2c, N3a, N3b) and N stage downstaging, with all p-values above 0.99.

Regarding pathological T stage, significant associations were found with downstaging. T2, T3, and T4a tumors had significantly higher odds of downstaging in both univariate and multivariate analyses. The univariate ORs were 2.769 (*p* = 0.030) for T2, 12.000 (*p* < 0.001) for T3, and 6.635 (*p* < 0.001) for T4a. In the multivariate analysis, the ORs remained significant: 2.581 (*p* = 0.045) for T2, 10.383 (*p* < 0.001) for T3, and 5.501 (*p* < 0.001) for T4a.

Grading did not show significant associations with N stage downstaging, with p-values of 0.187 for G2 and 0.418 for G3. Lymphatic invasion, perineural invasion, and resection margin were also not significantly associated with N stage downstaging, with p-values ranging from 0.123 to 0.367. However, vascular invasion showed a significant association in the univariate analysis (OR = 3.665, *p* = 0.035), though it did not reach significance in the multivariate analysis (OR = 2.504, *p* = 0.145).

### Univariate and multivariate analysis for overall survival

Univariate and multivariate analyses were conducted to identify significant predictors of OS in patients with OSCC. Regarding age, the univariate analysis suggested a trend toward worse survival in patients under 65 years (HR: 1.320, 95% confidence interval [CI]: 0.975–1.787, *p* = 0.072), although this was not statistically significant. Similarly, sex did not significantly affect survival (HR: 0.873, 95% CI: 0.637–1.194, *p* = 0.395). T stage concordance showed no significant survival impact in the univariate analysis, as patients with upstaged (HR: 1.010, 95% CI: 0.690–1.480, *p* = 0.958) or downstaged tumors (HR: 1.302, 95% CI: 0.895–1.894, *p* = 0.168) had no significant differences in survival compared to those with correctly staged tumors. However, N stage concordance significantly affected survival. In the univariate analysis, both upstaged (HR: 2.239, 95% CI: 1.601–3.123, *p* < 0.001) and downstaged N stages (HR: 2.247, 95% CI: 1.458–3.464, *p* < 0.001) were associated with poorer survival. In the multivariate analysis, upstaging remained a significant predictor of poor survival (HR: 1.653, 95% CI: 1.088–2.511, *p* = 0.018), while downstaging also continued to predict worse survival (HR: 1.948, 95% CI: 1.212–3.129, *p* = 0.006). Grading was another significant factor. In the univariate analysis, higher grading was associated with worse survival (HR: 1.497, 95% CI: 1.112–2.017, *p* = 0.008), and this association remained significant in the multivariate analysis (HR: 1.413, 95% CI: 1.013–1.971, *p* = 0.042), confirming that higher-grade tumors predict poorer survival outcomes. Lymphatic invasion showed a strong association with worse survival in the univariate analysis (HR: 2.116, 95% CI: 1.499–2.989, *p* < 0.001). However, in the multivariate analysis, the association weakened (HR: 1.554, 95% CI: 0.985–2.450, *p* = 0.058) and was no longer statistically significant. Vascular invasion also demonstrated an association with poorer survival in the univariate analysis (HR: 2.075, 95% CI: 1.190–3.619, *p* = 0.010), but this association was not significant in the multivariate analysis (HR: 0.910, 95% CI: 0.464–1.784, *p* = 0.783), suggesting that vascular invasion does not independently impact survival. Perineural invasion was associated with poorer survival in the univariate analysis (HR: 1.698, 95% CI: 1.201-2.400, *p* = 0.003), but this association diminished in the multivariate analysis (HR: 1.393, 95% CI: 0.963–2.014, *p* = 0.078). This finding suggests that, similar to vascular invasion, perineural invasion does not serve as an independent predictor of survival when adjusted for other variables. Finally, resection margin status was associated with survival in the univariate analysis (HR: 2.204, 95% CI: 1.383–3.513, *p* < 0.001), indicating that positive resection margins were associated with poorer survival. However, this association was no longer significant in the multivariate analysis (HR: 1.451, 95% CI: 0.777–2.710, *p* = 0.242), suggesting that resection margin status is not an independent predictor of survival when considering other factors. Please refer to Table S5 for further details.

### Univariate and multivariate analysis for recurrence-free survival

In the analysis of RFS, both univariate and multivariate analyses were conducted to identify significant clinicopathological predictors.

Age was not found to significantly affect RFS. In the univariate analysis, the HR for patients under 65 years compared to those aged 65 or older was 1.101 (95% CI: 0.758–1.601, *p* = 0.613), indicating no significant difference in RFS between these two age groups. Similarly, sex did not influence RFS, with the univariate analysis showing a HR of 1.075 (95% CI: 0.737–1.569, *p* = 0.707), suggesting no significant difference in survival between males and females.

T stage concordance did not significantly impact RFS. The univariate analysis revealed that upstaged cases had a HR of 1.056 (95% CI: 0.678–1.643, *p* = 0.810), and downstaged cases had a HR of 0.736 (95% CI: 0.426–1.271, *p* = 0.271), with neither result reaching statistical significance. Likewise, N stage concordance did not show a significant effect on RFS, with HRs of 1.352 (95% CI: 0.886–2.063, *p* = 0.162) for upstaged N stages and 1.260 (95% CI: 0.706–2.249, *p* = 0.435) for downstaged N stages.

Tumor grading was identified as a significant predictor of RFS. In the univariate analysis, higher grading was associated with poorer RFS, with a HR of 1.729 (95% CI: 1.203–2.485, *p* = 0.003). This remained significant in the multivariate analysis (HR: 1.603, 95% CI: 1.093–2.351, *p* = 0.016), confirming that grading is an independent predictor of RFS.

Lymphatic invasion also emerged as a significant predictor of RFS. In the univariate analysis, lymphatic invasion was associated with an increased risk of recurrence (HR: 2.033, 95% CI: 1.319–3.134, *p* = 0.001), and this remained significant in the multivariate analysis (HR: 1.917, 95% CI: 1.190–3.089, *p* = 0.007), confirming that lymphatic invasion independently predicts worse RFS.

In contrast, vascular invasion showed a significant association with worse RFS in the univariate analysis (HR: 2.297, 95% CI: 1.240–4.254, *p* = 0.008), but the effect was no longer significant in the multivariate analysis (HR: 1.334, 95% CI: 0.667–2.670, *p* = 0.415). Perineural invasion did not significantly affect RFS (HR: 1.433, 95% CI: 0.928–2.213, *p* = 0.104).

Finally, resection margin status was not a significant predictor of RFS (HR: 1.436, 95% CI: 0.652–3.160, *p* = 0.369), indicating that resection margin status does not impact RFS in this cohort. Please refer to Table S6 for further details.

### Overall survival

**Fig. 1:**
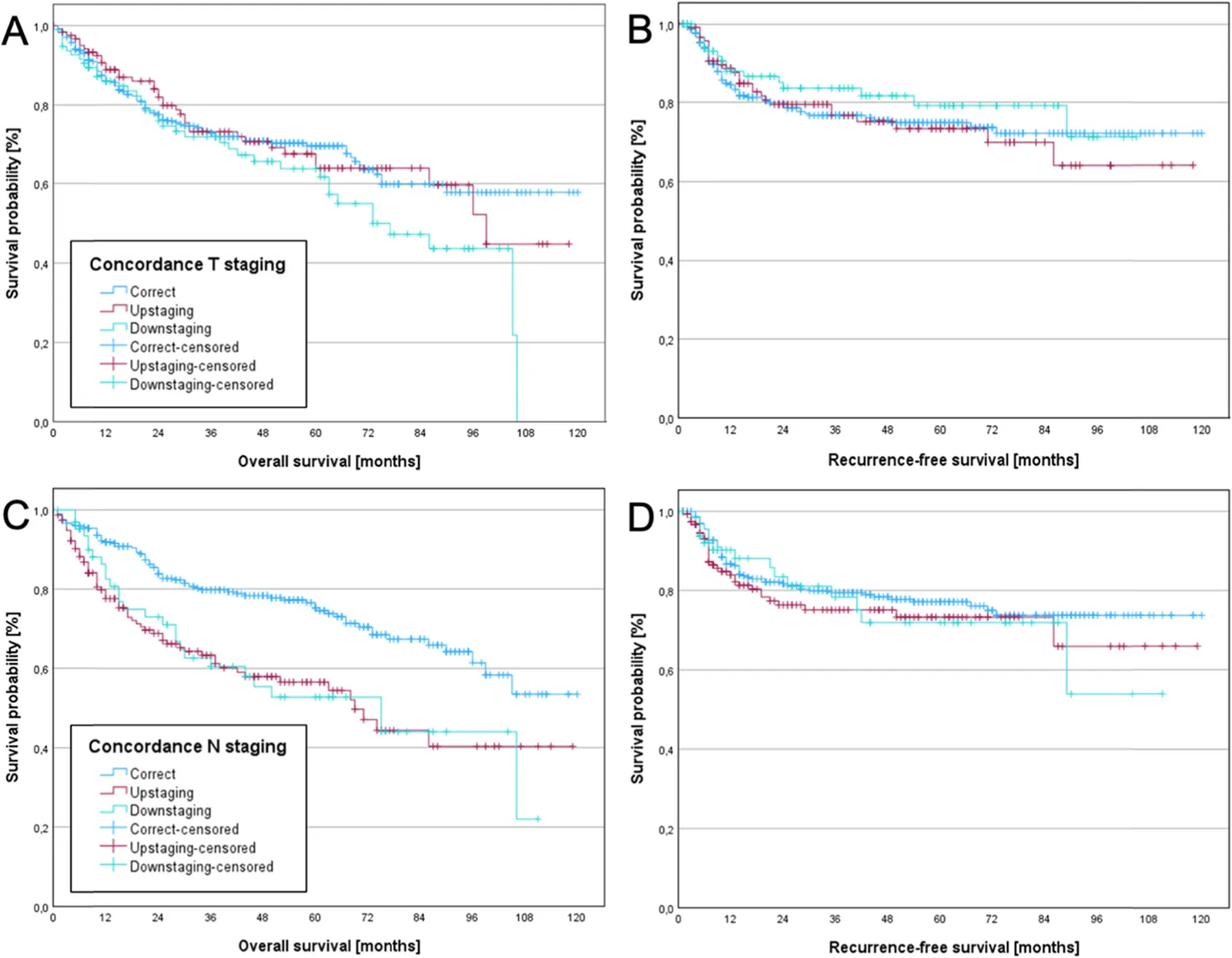
Kaplan-Meier survival curves stratified by concordance between clinical and pathological T and N categories. (**A**) *Overall Survival (T Staging)*: Patients downstaged in T staging after histopathological assessment exhibited a nonsignificant tendency toward reduced overall survival compared with correctly staged patients (log-rank, *p* = 0.078). No significant differences were observed between correctly staged patients and those upstaged after histopathological assessment (log-rank, *p* = 0.960) (**B**) Recurrence-free survival (T category): No pairwise comparison reached statistical significance (upstaged vs. downstaged: log-rank *p* = 0.318; downstaged vs. concordantly staged: log-rank *p* = 0.349) (**C**) *Overall Survival (N Staging)*: Both upstaged and downstaged groups showed significantly worse overall survival compared with correctly staged patients (log-rank, *p* < 0.001 and *p* = 0.001, respectively) (**D**) Recurrence-free survival (N category): No statistically significant difference was observed between upstaged and downstaged patients (log-rank *p* = 0.272)

In T staging, patients who were downstaged after histopathological assessment showed a nearly significant reduction in OS compared to those with correct staging (*p* = 0.078). However, no significant difference was found between the correctly staged and upstaged groups, as both groups exhibited similar OS rates (*p* = 0.960). Please refer to Fig. 1A for details.

In N staging, both the upstaged and downstaged groups demonstrated significantly worse OS compared to patients with correct staging. Specifically, upstaged N stages were associated with significantly reduced OS (*p* < 0.001), and downstaged N stages also showed significantly poorer OS (*p* = 0.001). Please see Fig. 1C for more information.

**Fig. 2:**
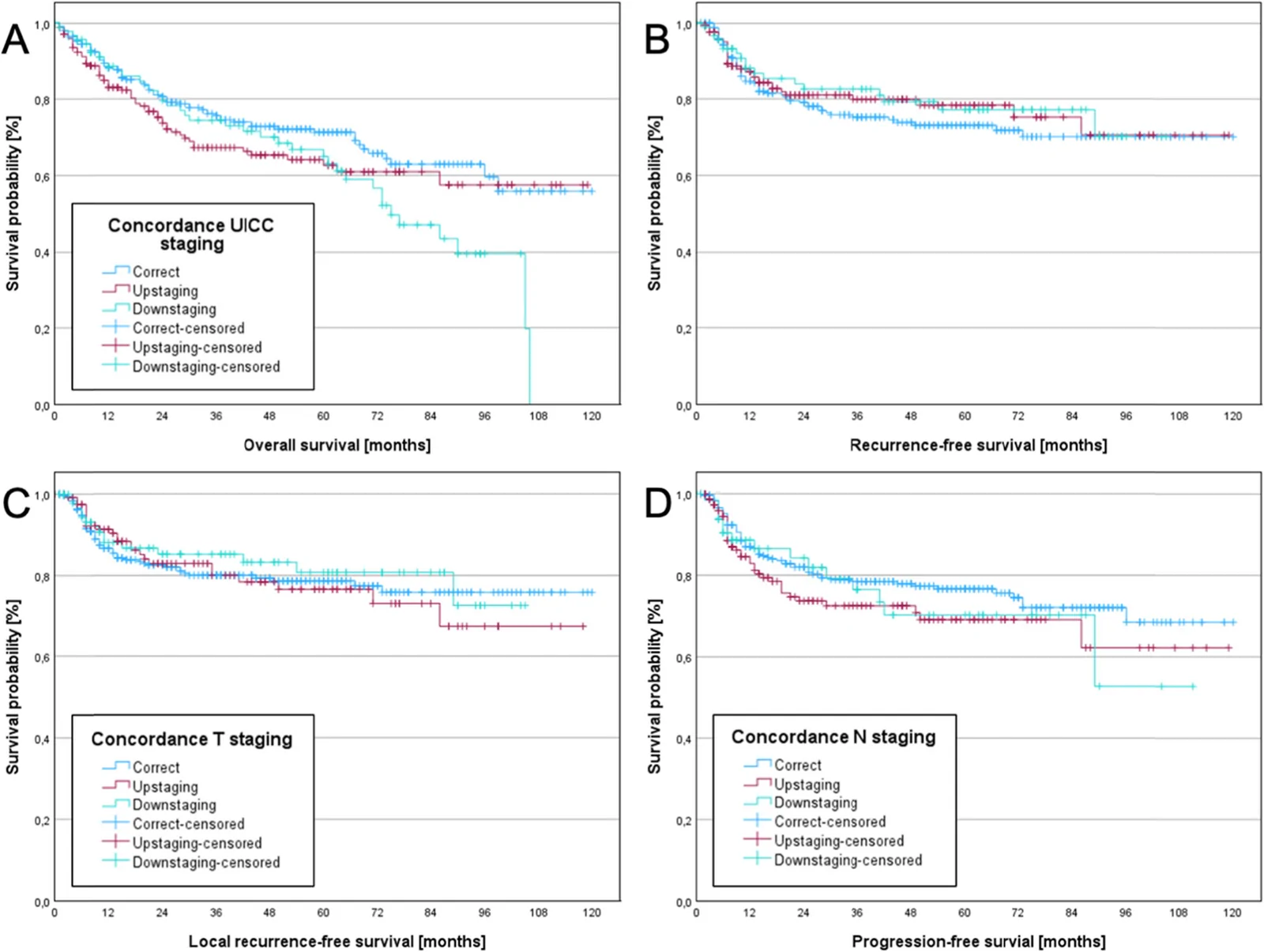
Kaplan–Meier survival curves stratified by concordance between clinical and pathological UICC, T, and N categories. (**A**) *Overall Survival (UICC Staging)*: Patients downstaged in UICC staging after histopathological assessment exhibited a significant reduction in overall survival compared with those correctly staged (log-rank, *p* = 0.046). No significant differences were observed in the other groups, though there was a nonsignificant tendency toward worse overall survival in upstaged patients compared with correctly staged patients (log-rank, *p* = 0.171) (**B**) *Recurrence-Free Survival (UICC Staging)*: No significant differences were observed between groups based on UICC staging (log-rank, *p* > 0.05) (**C**) *Local Recurrence-Free Survival (T Staging)*: No significant differences were observed between groups based on T staging (log-rank, *p* > 0.05) (**D**) Progression-free survival (N category): The comparison between upstaged and concordantly staged patients was not statistically significant (log-rank *p* = 0.138)

Regarding UICC staging, patients who were downstaged after histopathological assessment had significantly worse OS (*p* = 0.046) compared to those with correct staging. Conversely, no significant difference was observed between the upstaged and correctly staged groups, although there was a trend suggesting worse OS for the upstaged group (*p* = 0.171). Please refer to Fig. 2A for further details.

### Recurrence-free survival

No statistically significant differences in RFS were observed between groups based on T staging. However, a trend suggested worse survival outcomes in patients who were upstaged compared to those who were downstaged (*p* = 0.318). Additionally, a trend indicated improved RFS in patients who were downstaged after histopathological evaluation, compared to those who were accurately staged (*p* = 0.349). Please refer to Fig. 1B for further details.

Similarly, no significant differences in RFS were found between groups based on N staging. However, a trend indicated worse survival outcomes in patients who were upstaged compared to those who were downstaged (*p* = 0.272). Please see Fig. 1D for more information.

Lastly, no significant differences in RFS were identified between groups based on UICC staging (*p* > 0.05). Please refer to Fig. 2B for additional information.

### Local recurrence-free survival

No significant differences in LRFS were observed between the groups based on T staging (*p* > 0.05). Please refer to Fig. 2C for further details.

### Progression-free survival

No significant differences in PFS were observed between the groups based on N staging. Patients with N-stage upstaging showed a trend toward poorer progression-free survival compared with correctly staged patients (*p* = 0.138), although this difference did not reach statistical significance. Likewise, no statistically significant differences were observed between the remaining staging groups. Please refer to Fig. 2D.

## Discussion

Accurate clinical staging of OSCC is essential for treatment planning and prognostic assessment and is becoming increasingly important in the context of neoadjuvant treatment and de-escalated neck management strategies [8–10]. In the present study, concordance between clinical and pathological staging was moderate for both T and N categories, with discordance occurring in a substantial proportion of patients. In addition, several clinicopathological characteristics were associated with staging discordance, and N-category discordance was associated with overall survival.

### Concordance in T- and N-staging

Concordance between clinical and pathological T staging varied considerably across clinical T categories. Accuracy was highest for cT1 (68.32%) and cT4a tumors (76.55%), whereas only approximately 40% of cT2 and cT3 tumors were correctly staged, with both under- and overdiagnosis occurring frequently. These discrepancies reflect the limitations of clinical examination and imaging in accurately determining tumor extent. Assessment may be particularly challenging depending on tumor localization, pain, or pharyngeal reflex [4], while peritumoral oedema on CT may lead to overestimation of tumor extent. Such inaccuracies are clinically relevant because overdiagnosis may expose patients to unnecessary treatment-related morbidity and impaired quality of life [11, 12].

N staging showed similar variability. Although cN0 had the highest concordance (74.21%), approximately one quarter of clinically node-negative patients were pathologically upstaged, indicating occult nodal disease. Concordance was substantially lower in clinically node-positive categories, emphasizing the limitations of current clinical tools in assessing nodal involvement.

Overdiagnosis may be due to non-malignant causes of lymph node swelling or limitations in imaging techniques like CT and MRI [15]. Additionally, as previously reported by our group, variations in the criteria used to define pathological lymph nodes on CT imaging may significantly influence the accuracy of clinical staging [6]. Clinical lymph node assessment during physical examination is also difficult in patients with obesity because the possibility of manual palpation is limited [4]. Advanced imaging, such as 18 F-FDG-PET/CT, could improve detection of lymph node metastases by identifying metabolic differences between healthy and tumor tissue [16, 17].

Our findings are broadly consistent with previous studies. Koch et al. reported concordance rates of 52.2% for T staging, 53.5% for N staging, and 54.9% for overall TNM classification in a multicenter cohort of 560 patients with HNSCC [18].

Similarly, Kreppel et al. reported concordance rates of 62% for T and 59% for N staging in 392 treatment-naive patients with OSCC. In contrast to our cohort, however, clinical overstaging of both the primary tumor and nodal disease was more frequent in their study [19]. Kilic et al., analyzing data from the National Cancer Database, reported an overall concordance of 67.3% and found N-stage discordance to be slightly more frequent than T-stage discordance, with N upstaging occurring more commonly than downstaging [20]. Henriques et al. likewise observed substantial staging discrepancies and, in line with our findings, reported the highest N-stage concordance for cN0 and lower concordance for cN1 and cN2a disease [21]. Sousa et al. reported pathological N upstaging in 34.3% of cases as well as discrepancies in T staging [22], whereas Choi et al. found considerably higher concordance rates of 87.3% for T and 82.5% for N staging [23].

Other studies further illustrate the heterogeneity of reported staging accuracy. Tariq et al. found only 40% concordance for nodal staging and 35% agreement between overall cTNM and pTNM in a smaller cohort of 80 patients [5]. Gupta et al. reported the highest T-stage concordance for cT1 and cT4 and the highest N-stage concordance for cN0 and cN3 in 127 patients with OSCC [3]. Hondorp et al., studying 580 surgically treated patients with HNSCC, observed staging discordance in 33% of cases with an overall Cohen’s kappa of 0.55 [2]. In their study discordance was particularly pronounced for T2 OSCC and N1 disease, whereas several other site- and stage-specific categories showed substantially higher agreement, highlighting the variability of staging accuracy according to tumor site and stage [2].

### Factors influencing concordance

Among the clinicopathological characteristics examined, tumor grading, lymphatic invasion, and perineural invasion were associated with staging discordance, whereas age and sex were not.

The regression analyses provided a more differentiated assessment of factors associated with T- and N-category up- and downstaging.

For T-stage upstaging, poor differentiation (G3) and perineural invasion remained significant in multivariable analysis, whereas cT4a tumors were less likely to be upstaged. These findings suggest that adverse histopathological characteristics may be associated with clinical underestimation of primary tumor extent.

For T-stage downstaging, the initial clinical T category was the predominant associated factor, with cT2, cT3, and cT4a tumors more frequently downstaged than cT1 tumors. This finding should also be interpreted in light of the fact that the opportunity for downstaging necessarily increases with higher initial clinical categories.

Biron et al. similarly reported pathological upstaging among clinically early-stage tumors, whereas downstaging occurred in a proportion of clinically advanced-stage tumors [4].

For N-stage upstaging, cN1 and cN2b categories remained significantly associated with upstaging in multivariable analysis. Importantly, lymphatic and perineural invasion were also independent predictors of N-stage upstaging, whereas grading and vascular invasion did not remain significant after multivariable adjustment.

N-stage downstaging was mainly associated with pathological T category, with pT2, pT3, and pT4a tumors showing higher odds of downstaging. The other investigated clinicopathological variables did not remain significantly associated in multivariable analysis.

These findings indicate that staging discordance is associated with both tumor extent and adverse histopathological characteristics. Lin et al., in a large cohort of patients with OSCC, likewise identified higher pathological T stage as a risk factor for N-stage downstaging and reported additional associations with betel chewing, depth of invasion ≥ 10 mm, and perineural invasion [1].

In addition, we previously demonstrated that lymphatic and perineural invasion are associated with an increased risk of occult cervical lymph node metastases, providing a potential explanation for the observed association of these features with pathological N-category up-categorization [24].

Another point that should be considered is that tumor shrinkage after surgical resection and during fixation may have influenced the comparison between clinical and pathological T stage. This effect may contribute to apparent clinical overestimation of tumor extent and subsequent pathological downstaging. However, the degree of tissue shrinkage is variable and depends on tumor localization, tissue composition, specimen handling, fixation, and pathological processing. Therefore, applying a uniform correction factor retrospectively was not feasible and may have introduced additional bias.

Overall, our findings suggest that histopathological features already identified in the preoperative biopsy may provide complementary information regarding the risk of clinical understaging. In addition, Na’ara et al. proposed incorporating perineural invasion into the T-classification system and demonstrated improved prognostic performance compared with T classification alone [25]. Moreover, physicians should be aware that patients with tumors exhibiting such histological features may be at increased risk of pathological up-categorization. In our cohort, adverse features such as poor differentiation and perineural invasion were associated with subsequent pathological up-categorization. If identified preoperatively, such findings may therefore alert the treating team to an increased risk of clinical understaging and support particularly thorough preoperative assessment.

### Survival outcomes

T-category discordance was not significantly associated with OS or RFS in our cohort. Although T-stage downstaging showed a trend toward reduced OS, this did not reach statistical significance.

In contrast, both N-category upstaging and downstaging were associated with worse OS, emphasizing the prognostic relevance of discrepancies in nodal assessment. However, this association should not be interpreted as evidence of a causal effect of staging discordance itself. Similar observations of worse survival among overstaged patients in other cancers suggest that such discrepancies may also reflect confounding by tumor burden, comorbidities, second primary malignancies, or competing causes of death [26].

Overall, our findings align with some previous studies while differing from others. For instance, Choi et al. reported no significant differences in recurrence rates or OS between patients with upstaged and unchanged clinical staging. They concluded that clinical staging discrepancies did not significantly influence prognosis when standard treatment protocols were adhered to [23]. In contrast, and consistent with our findings, Kreppel et al. identified a significant difference in OS between correctly staged and understaged patients with respect to N staging [19]. Sousa et al. observed that only patients with pN upstaging exhibited significantly worse survival (*p* = 0.005), while discrepancies in T staging had no statistically significant impact on survival or recurrence rates (*p* > 0.05) [22]. This is partly consistent with our findings: T-category discordance was not associated with either OS or recurrence-free survival, whereas N-category discordance was associated with OS. Notably, in our cohort, both N-category up-categorization and down-categorization were significantly associated with worse OS. Biron et al., in contrast, found no significant differences in disease-specific survival between patients with upstaged, downstaged, or unchanged staging in a multi-institutional cohort of 199 patients [4].

Clinically occult nodal disease may contribute to the adverse outcome among patients with N-category up-categorization, whereas the poorer OS in down-categorized patients may reflect residual confounding, including primary tumor burden, comorbidities, inflammatory nodal enlargement, second primary malignancies, or competing causes of death.

### Limitations of the study

Several limitations should be acknowledged in this study. First, while we focused on T and N staging discrepancies, an analysis of M stage discordance was not feasible due to the small number of patients with distant metastases. In addition, the exact metric distance of the closest resection margin was not consistently documented in a standardized manner in all historical pathology reports. Therefore, a reliable retrospective reclassification into clear margins ( > = 5 mm), close margins (< 5 mm), and positive margins was not possible for the entire cohort. In addition, although DOI and ENE were frequently documented, this information was not available for all patients due to the retrospective nature of the study. Moreover, our study was observational in nature, and other variables such as comorbidities and other patient-specific factors could have influenced survival outcomes. Another limitation of this study is that clinical staging was based on physical examination and contrast-enhanced CT as the standardized imaging modality. This approach is consistent with the German S3 guideline for OSCC [14]. However, MRI may provide superior soft-tissue contrast in selected subsites, particularly in tongue carcinomas, tumors with suspected intramuscular or perineural spread, and tumors close to the masticator space or skull base. Furthermore, FDG-PET/CT was not systematically performed to exclude distant metastases. Finally, while we focused on clinical versus pathological staging, we did not explore the impact of molecular or genetic markers on prognosis, which may provide additional insight into the accuracy of staging and survival prediction in OSCC. However, it must be noted that in our study, all patients were uniformly classified according to the 8th edition of the UICC classification, which may represent one of the study’s strengths. In addition, although we had to exclude a number of patients due to missing data, we were able to include a larger cohort of patients compared to previous studies. This allowed us to identify trends and significant findings that had not been observed before.

### Conclusion

In conclusion, as neoadjuvant therapy and de-escalated neck management strategies, such as SLNB, become increasingly integrated into the management of OSCC, accurate clinical staging is of paramount importance. Further refinement of diagnostic tools and staging strategies is needed to minimize clinically relevant discrepancies. Histopathological adverse features already identified in preoperative biopsies may additionally help identify patients at increased risk of clinical understaging and support a more thorough preoperative assessment.

## Supplementary Information

Below is the link to the electronic supplementary material.

Supplementary Material 1 (DOCX 33.3 KB)

## Data Availability

The data that support the findings of this study are available from the corresponding author upon reasonable request.

## Abbreviations

cN: Clinical nodal staging
cT: Clinical tumor staging
CT: Computed tomography
FDG-PET/CT: Fluorodeoxyglucose positron emission tomography/computed tomography
HNSCC: Head and neck squamous cell carcinoma
HR: Hazard ratio
LRFS: Local recurrence-free survival
M stage: Distant metastases staging
MRI: Magnetic resonance imaging
N stage: Nodal staging
OR: Odds ratio
OSCC: Oral squamous cell carcinoma
OS: Overall survival
PFS: Progression-free survival
pN: Pathological nodal staging
pT: Pathological tumor staging
RFS: Recurrence-free survival
SLNB: Sentinel lymph node biopsy
T stage: Tumor staging
TNM: Tumor, node, metastasis (staging system)
UICC: Union for international cancer control
