# Supplementary material for: Clinical vs. pathological staging in oral squamous cell carcinoma: frequency of misjudgment, associated risk factors, and impact on survival outcomes
Source: Clin Oral Investig. 2026 Nov 14;30(12):578. doi: 10.1007/s00784-026-07275-y (PMC13769718; doi:10.1007/s00784-026-07275-y)
Supplement: Supplementary file 1 [file 784_2026_7275_MOESM1_ESM.docx]

**Supplemental to:**

**Clinical vs. Pathological Staging in Oral Squamous Cell Carcinoma: Frequency of Misjudgment, Associated Risk Factors, and Impact on Survival Outcomes**

Ann-Kristin Struckmeier MD, DMD, Martin Gosau MD, DMD, Christian Betz MD,

Waldemar Wilczak MD, Ralf Smeets MD, DMD

**Table S1.** Univariate and multivariate analysis of the risk factors for upstaging T stage after histopathological assessment

| **Clinicopathological characteristics** | **Univariate analysis** | | | **Multivariate analysis** | | | | |
| --- | --- | --- | --- | --- | --- | --- | --- | --- |
|  | **OR** | **95% CI** | **p value** | **OR** | **95% CI** | | **p value** | |
| **Age: < 65 vs. ≥ 65 years** | 1.115 | 0.733-1.696 | 0.610 |  | | | | |
| **Sex** | 0.977 | 0.646-1.479 | 0.912 |  |  |  |  |  |
| **Clinical T stage** | | | | | | | | |
| **T1** | 1 | | | 1 | | | | |
| **T2** | 1.824 | 1.137-2.925 | 0.013* | 1.273 | | 0.745-2.175 | | 0.377 |
| **T3** | 1.553 | 0.690-3.495 | 0.287 | 0.886 | | 0.369-2.123 | | 0.785 |
| **T4a** | 0.020 | 0.003-0.146 | <0.001* | 0.012 | | 0.002-0.093 | | <0.001* |
| **T4b** | 0.000 | 0.000 | 0.999 | 0.000 | | 0.00 | | 0.999 |
| **Pathological N stage** | | | |  | | | | |
| **pN0** | 1 | | |  |  |  |  |  |
| **pN1** | 1.242 | 0.670-2.301 | 0.492 |  |  |  |  |  |
| **pN2a** | 1.053 | 0.325-3.418 | 0.931 |  |  |  |  |  |
| **pN2b** | 1.609 | 0.808-3.205 | 0.176 |  |  |  |  |  |
| **pN2c** | 1.738 | 0.727-4.145 | 0.214 |  |  |  |  |  |
| **pN3a** | 0.000 | 0.000 | 1.000 |  |  |  |  |  |
| **pN3b** | 1.449 | 0.687-3.054 | 0.330 |  |  |  |  |  |
| **Grading** | | | |  |  |  |  |  |
| **G1** | 1 | | | 1 | | | | |
| **G2** | 2.808 | 0.820-9.613 | 0.100 | 2.603 | 0.714-9.494 | | 0.147 | |
| **G3** | 4.823 | 1.357-17.139 | 0.015* | 4.483 | 1.154-17.423 | | 0.030* | |
| **Lymphovascular invasion** | 1.488 | 0.900-2.458 | 0.121 |  | | | | |
| **Vascular invasion** | 1.492 | 0.653-3.409 | 0.343 |  |  |  |  |  |
| **Perineural invasion** | 2.079 | 1.303-3.317 | 0.002* | 2.459 | 1.390-4.350 | | 0.002* | |
| **Resection margin** | 0.545 | 0.176-1.686 | 0.292 |  | | | | |

**Abbreviations:** N stage = nodal stage, T stage = tumor stage

A p value < 0.05 was considered statistically significant. Statistically significant differences are marked with an asterisk.

**Table S2.** Univariate and multivariate analysis of the risk factors for downstaging T stage after histopathological assessment

| **Clinicopathological characteristics** | **Univariate analysis** | | |
| --- | --- | --- | --- |
|  | **OR** | **95% CI** | **p value** |
| **Age: < 65 vs. ≥ 65 years** | 0.941 | 0.602-1.471 | 0.789 |
| **Sex** | 0.991 | 0.629-1.585 | 0.696 |
| **Clinical T stage** | | | |
| **T1** | 1 | | |
| **T2** | 78.158 | 10.583-577.234 | <0.001* |
| **T3** | 90.588 | 11.181-733.913 | <0.001* |
| **T4a** | 32.703 | 4.396-243.300 | <0.001* |
| **T4b** | 0.000 | 0.000 | <0.001* |
| **Pathological N stage** | | | |
| **pN0** | 1 | | |
| **pN1** | 1.415 | 0.767-2.608 | 0.266 |
| **pN2a** | 0.284 | 0.036-2.245 | 0.233 |
| **pN2b** | 0.579 | 0.214-1.567 | 0.282 |
| **pN2c** | 0.417 | 0.093-1.873 | 0.254 |
| **pN3a** | 3.127 | 0.193-50.722 | 0.423 |
| **pN3b** | 1.433 | 0.665-3.089 | 0.358 |
| **Grading** | | | |
| **G1** | 1 | | |
| **G2** | 0.840 | 0.371-1.900 | 0.675 |
| **G3** | 0.783 | 0.310-1.978 | 0.605 |
| **Lymphovascular invasion** | 0.571 | 0.285-1.142 | 0.113 |
| **Vascular invasion** | 0.236 | 0.032-1.757 | 0.159 |
| **Perineural invasion** | 0.885 | 0.491-1.595 | 0.685 |
| **Resection margin** | 0.466 | 0.120-1.806 | 0.269 |

**Abbreviations:** N stage = nodal stage, T stage = tumor stage

A p value < 0.05 was considered statistically significant. Statistically significant differences are marked with an asterisk.

**Table S3.** Univariate and multivariate analysis of the risk factors for upstaging N stage after histopathological assessment

| **Clinicopathological characteristics** | **Univariate analysis** | | | **Multivariate analysis** | | | | |
| --- | --- | --- | --- | --- | --- | --- | --- | --- |
|  | **OR** | **95% CI** | **p value** | **OR** | **95% CI** | | **p value** | |
| **Age: < 65 vs. ≥ 65 years** | 1.038 | 0.708-1.521 | 0.848 |  | | | | |
| **Sex** | 1.048 | 0.711-1.545 | 0.811 |  |  |  |  |  |
| **Clinical N stage** | | | | | | | | |
| **N0** | 1 | | | 1 | | | | |
| **N1** | 4.316 | 2.344-7.949 | <0.001* | 2.361 | | 1.090-5.113 | | 0.029* |
| **N2a** | 2.878 | 0.706-11.725 | 0.140 | 4.017 | | 0.614-26.276 | | 0.147 |
| **N2b** | 9.592 | 3.744-24.575 | <0.001* | 5.667 | | 1.866-17.209 | | 0.002* |
| **N2c** | 2.055 | 0.638-6.625 | 0.228 | 0.729 | | 0.157-3.386 | | 0.686 |
| **N3a** | 0.00 | 0.00 | 1.00 | 0.00 | | 0.00 | | 1.000 |
| **N3b** | 0.00 | 0.00 | 1.00 | 0.00 | | 0.00 | | 1.000 |
| **Pathological T stage** | | | | | | | | |
| **T1** | 1 | | | 1 | | | | |
| **T2** | 2.677 | 1.548-4.629 | <0.001* | 2.183 | | 1.119-4.258 | | 0.022* |
| **T3** | 5.786 | 2.096-11.520 | <0.001* | 3.029 | | 1.335-6.872 | | 0.008* |
| **T4a** | 3.602 | 2.118-6.123 | <0.001* | 1.650 | | 0.826-3.299 | | 0.156 |
| **T4b** | 0.000 | 0.000 | 0.999 | 0.00 | | 0.00 | | 0.999 |
| **Grading** | | | | | | | | |
| **G1** | 1 | | | 1 | | | | |
| **G2** | 11.239 | 1.501-84.124 | 0.018* | 0.00 | 0.00 | | 0.998 | |
| **G3** | 24.537 | 3.208-187.649 | 0.002* | 0.00 | 0.00 | | 0.998 | |
| **Lymphovascular invasion** | 7.702 | 4.572-12.973 | <0.001* | 6.017 | | 3.095-11.689 | | <0.001* |
| **Vascular invasion** | 5.860 | 2.070-16.592 | <0.001* | 1.551 | | 0.408-5.889 | | 0.519 |
| **Perineural invasion** | 7.541 | 4.325-12.122 | <0.001* | 1.838 | 1.063-3.179 | | 0.029* | |
| **Resection margin** | 1.452 | 0.592-3.564 | 0.415 |  | | | | |

**Abbreviations:** N stage = nodal stage, T stage = tumor stage

A p value < 0.05 was considered statistically significant. Statistically significant differences are marked with an asterisk.

**Table S4.** Univariate and multivariate analysis of the risk factors for downstaging N stage after histopathological assessment

| **Clinicopathological characteristics** | **Univariate analysis** | | | **Multivariate analysis** | | | | |
| --- | --- | --- | --- | --- | --- | --- | --- | --- |
|  | **OR** | **95% CI** | **p value** | **OR** | **95% CI** | | **p value** | |
| **Age: < 65 vs. ≥ 65 years** | 0.755 | 0.443-1.286 | 0.301 |  | | | | |
| **Sex** | 0.893 | 0.519-1.537 | 0.683 |  |  |  |  |  |
| **Clinical N stage** | | | |  |  |  |  |  |
| **N0** | 1 | | |  |  |  |  |  |
| **N1** | 0.000 | 0.000 | 0.993 |  |  |  |  |  |
| **N2a** | 0.000 | 0.000 | 0.993 |  |  |  |  |  |
| **N2b** | 0.000 | 0.000 | 0.992 |  |  |  |  |  |
| **N2c** | 0.000 | 0.000 | 0.993 |  |  |  |  |  |
| **N3a** | 0.000 | 0.000 | 0.999 |  |  |  |  |  |
| **N3b** | 0.000 | 0.000 | 0.993 |  |  |  |  |  |
| **Pathological T stage** | | | | | | | | |
| **T1** | 1 | | | 1 | | | | |
| **T2** | 2.769 | 1.101-6.964 | 0.030* | 2.581 | | 1.022-6.517 | | 0.045* |
| **T3** | 12.000 | 4.610-31.235 | <0.001* | 10.383 | | 3.975-27.118 | | <0.001* |
| **T4a** | 6.635 | 2.899-15.182 | <0.001* | 5.501 | | 2.380-12.781 | | <0.001* |
| **Grading** | | | |  | | | | |
| **G1** | 1 | | |  |  |  |  |  |
| **G2** | 0.573 | 0.251-1.311 | 0.187 |  |  |  |  |  |
| **G3** | 0.669 | 0.253-1.770 | 0.418 |  |  |  |  |  |
| **Lymphovascular invasion** | 1.475 | 0.634-3.432 | 0.367 |  |  |  |  |  |
| **Vascular invasion** | 3.665 | 1.096-12.264 | 0.035* | 2.504 | | 0.728-8.606 | | 0.145 |
| **Perineural invasion** | 1.497 | 0.788-2.844 | 0.218 |  | | | | |
| **Resection margin** | 2.173 | 0.810-5.831 | 0.123 |  |  |  |  |  |

**Abbreviations:** N stage = nodal stage, T stage = tumor stage

A p value < 0.05 was considered statistically significant. Statistically significant differences are marked with an asterisk.

**Table S5.** Univariate and multivariate analysis of clinicopathological factors affecting overall survival in oral squamous cell carcinoma

| **Clinicopathological characteristics** | **Univariate analysis** | | | **Multivariate analysis** | | |
| --- | --- | --- | --- | --- | --- | --- |
|  | **HR** | **95% CI** | **p value** | **HR** | **95% CI** | **p value** |
| **Age: < 65 vs. ≥ 65 years** | 1.320 | 0.975-1.787 | 0.072 |  | | |
| **Sex** | 0.873 | 0.637-1.194 | 0.395 |  |  |  |
| **Concordance T stages** | | | |  |  |  |
| **Correctly diagnosed** | 1 | | |  |  |  |
| **Upstaged** | 1.010 | 0.690-1.480 | 0.958 |  |  |  |
| **Downstaged** | 1.302 | 0.895-1.894 | 0.168 |  |  |  |
| **Concordance N stages** | | | | | | |
| **Correctly diagnosed** | 1 | | | 1 | | |
| **Upstaged** | 2.239 | 1.601-3.123 | <0.001* | 1.653 | 1.088-2.511 | 0.018* |
| **Downstaged** | 2.247 | 1.458-3.464 | <0.001* | 1.948 | 1.212-3.129 | 0.006* |
| **Grading** | 1.497 | 1.112-2.017 | 0.008* | 1.413 | 1.013-1.971 | 0.042* |
| **Lymphovascular invasion** | 2.116 | 1.499-2.989 | <0.001* | 1.554 | 0.985-2.450 | 0.058 |
| **Vascular invasion** | 2.075 | 1.190-3.619 | 0.010* | 0.910 | 0.464-1.784 | 0.783 |
| **Perineural invasion** | 1.698 | 1.201-2.400 | 0.003* | 1.393 | 0.963-2.014 | 0.780 |
| **Resection margin** | 2.204 | 1.383-3.513 | <0.001* | 1.451 | 0.777-2.710 | 0.242 |

**Abbreviations:** N stage = nodal stage, T stage = tumor stage

A p value < 0.05 was considered statistically significant. Statistically significant differences are marked with an asterisk.

**Table S6.** Univariate and multivariate analysis of clinicopathological factors affecting recurrence-free survival in oral squamous cell carcinoma

| **Clinicopathological characteristics** | **Univariate analysis** | | | **Multivariate analysis** | | |
| --- | --- | --- | --- | --- | --- | --- |
|  | **HR** | **95% CI** | **p value** | **HR** | **95% CI** | **p value** |
| **Age: < 65 vs. ≥ 65 years** | 1.101 | 0.758-1.601 | 0.613 |  | | |
| **Sex** | 1.075 | 0.737-1.569 | 0.707 |  |  |  |
| **Concordance T stages** | | | |  |  |  |
| **Correctly diagnosed** | 1 | | |  |  |  |
| **Upstaged** | 1.056 | 0.678-1.643 | 0.810 |  |  |  |
| **Downstaged** | 0.736 | 0.426-1.271 | 0.271 |  |  |  |
| **Concordance N stages** | | | |  |  |  |
| **Correctly diagnosed** | 1 | | |  |  |  |
| **Upstaged** | 1.352 | 0.886-2.063 | 0.162 |  |  |  |
| **Downstaged** | 1.260 | 0.706-2.249 | 0.435 |  |  |  |
| **Grading** | 1.729 | 1.203-2.485 | 0.003* | 1.603 | 1.093-2.351 | 0.016* |
| **Lymphovascular invasion** | 2.033 | 1.319-3.134 | 0.001* | 1.917 | 1.190-3.089 | 0.007* |
| **Vascular invasion** | 2.297 | 1.240-4.254 | 0.008* | 1.334 | 0.667-2.670 | 0.415 |
| **Perineural invasion** | 1.433 | 0.928-2.213 | 0.104 |  | | |
| **Resection margin** | 1.436 | 0.652-3.160 | 0.369 |  |  |  |

**Abbreviations:** N stage = nodal stage, T stage = tumor stage

A p value < 0.05 was considered statistically significant. Statistically significant differences are marked with an asterisk.
